# Fully Integrated Wearable Device for Continuous Sweat Lactate Monitoring in Sports

**DOI:** 10.1021/acssensors.3c00708

**Published:** 2023-06-08

**Authors:** Xing Xuan, Chen Chen, Agueda Molinero-Fernandez, Emil Ekelund, Daniele Cardinale, Mikael Swarén, Lars Wedholm, Maria Cuartero, Gaston A. Crespo

**Affiliations:** † Department of Chemistry, KTH Royal Institute of Technology, Teknikringen 30, SE-100 44 Stockholm, Sweden; ‡ UCAM-SENS, Universidad Católica San Antonio de Murcia, UCAM HiTech, Avda. Andres Hernandez Ros 1, 30107 Murcia, Spain; § Department of Physiology, Nutrition, and Biomechanics, The Swedish School of Sport and Health Sciences, GIH, SE-11486 Stockholm, Sweden; ∥ Swedish Unit of Metrology in Sports, Institution of Health and Welfare, Dalarna University, SE-791 88 Falun, Sweden; ⊥ Institution of Health and Welfare, Dalarna University, SE-791 88 Falun, Sweden

**Keywords:** chemical digitization, sweat lactate, wearable sensing interfaces, outer diffusion-limiting membrane, sensing device

## Abstract

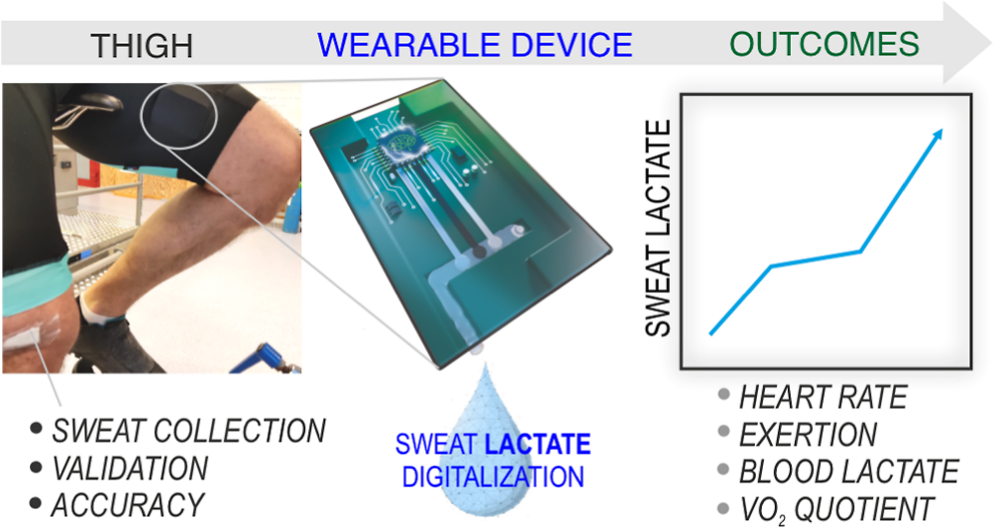

The chemical digitalization of sweat using wearable sensing interfaces is an attractive alternative to traditional blood-based protocols in sports. Although sweat lactate has been claimed to be a relevant biomarker in sports, an analytically validated wearable system to prove that has not yet been developed. We present a fully integrated sweat lactate sensing system applicable to in situ perspiration analysis. The device can be conveniently worn in the skin to monitor real-time sweat lactate during sports, such as cycling and kayaking. The novelty of the system is threefold: advanced microfluidics design for sweat collection and analysis, an analytically validated lactate biosensor based on a rational design of an outer diffusion-limiting membrane, and an integrated circuit for signal processing with a custom smartphone application. The sensor covering the range expected for lactate in sweat (1–20 mM), with appropriate sensitivity (−12.5 ± 0.53 nA mM^–1^), shows an acceptable response time (<90 s), and the influence of changes in pH, temperature, and flow rate are neglectable. Also, the sensor is analytically suitable with regard to reversibility, resilience, and reproducibility. The sensing device is validated through a relatively high number of on-body tests performed with elite athletes cycling and kayaking in controlled environments. Correlation outcomes between sweat lactate and other physiological indicators typically accessible in sports laboratories (blood lactate, perceived exhaustion, heart rate, blood glucose, respiratory quotient) are also presented and discussed in relation to the sport performance monitoring capability of continuous sweat lactate.

Over the past few decades, lactate has become an important analytical target in several fields, including the food industry, clinical diagnostics, and sport science.^1^ In the last decade, the increase of blood lactate levels during physical exercise has been employed to assess the performance of athletes using the lactate threshold (LT) calculation.^2^ For this purpose, lactate information is obtained by discrete measurements (every 5–20 min) using a traditional finger-prick blood gadget, which is, to some extent, painful and stressful and, more importantly, interrupts athletic activity. Moreover, this strategy provides discrete-type data; hence, it may lose valuable physiological information and prevent rapid feedback to the athletes. A less invasive approach allowing for continuous and real-time lactate monitoring is in high demand by sports physiology scientists, athletes, and coaches.

Sweat offers a promising alternative to blood for daily analysis performed on the sports field. Sweat is naturally generated during any physical activity, collection is non-invasive, and it contains abundant biomarkers related to body conditions, such as lactate.^3^ Despite recent studies concluding that sweat lactate is a strong candidate to replace blood analysis for sports performance evaluation,^4,5^ this remains controversial. In essence, until the time of writing, all conclusions about sweat lactate have been drawn from data generated in very distinct sample-based approaches, which are subject to significant errors.^6^ Thus, studies have shown contradictory tendencies for sweat lactate: it increases or decreases with activity evolution, and the existence and absence of correlation with blood lactate.

Several lactate sensors have been reported in the literature attempting to provide trusted on-body data for physiological evidence, including colorimetric, electrochemiluminescent, and electrochemical readouts.^7−10^ Promphet et al. have developed a colorimetric textile-based lactate sensor for on-body measurements.^7^ The sensor is simple, low cost, and easily fabricated and showed an acceptable linear response range (LRR) from 1 to 25 mM. However, neither the validation of on-body tests nor the effect of sweat accumulation in the calculated lactate levels was addressed (the device is not a microfluidic one). In addition, the analysis was discrete rather than continuous and required a camera and a portable spectrophotometer. Chen et al. have proposed a flexible electrochemiluminescence platform with a range of response up to 20 mM.^11^ This sensor was successfully applied in two on-body tests, and some discrete values were obtained. Nevertheless, the degradation and/or leaching of the luminophore material during long-term measurements is a concern, as it may lead to inaccurate results over time.

Electrochemical sensors are one of the most powerful analytical tools for continuous monitoring of sweat lactate, not only in terms of simplicity, low cost, portability, and easy miniaturization but also in their analytical performance.^9,12−15^ These sensors primarily rely on enzymes, such as lactate oxidase,^16−19^ in combination with redox mediators (e.g., Prussian blue),^9,14^ and employ amperometry for detection. Importantly, most of these sensors are sensitive to physiological variables (e.g., pH and temperature) and have stability issues, which require correction before the final lactate levels can be provided.^14^ Potentiometry^20^ and electrochemical impedance spectroscopy^21^ have been proposed to avoid using a redox mediator; conductive polymers have been used to develop non-enzymatic sensors;^20^ and the protective layers (e.g., hydrogels) or polymeric membranes^22^ have been added to improve the overall stability and lifetime. Recently, our group has demonstrated that using lipophilic outer membranes significantly enhances not only the stability but also the LRR of enzymatic lactate biosensors.^9,23^

In recent studies, the development of sweat lactate electrochemical sensors has been combined with advanced sampling approaches (i.e., microfluidics design) and the integration of electronic hardware and software. For example, Komkova et al. have reported an enzyme-based lactate biosensor equipped with a capillary casing, as the microfluidics system, and wireless electronics.^24^ The device was tested on healthy volunteers engaged in running and demonstrated suitable agreement with standard analytical techniques. Table S1 in the Supporting Information summarizes the characteristics of some electrochemical sensors for sweat lactate detection in terms of analytical performance, level of integration, sampling strategy, validation methodology, physiological evaluation, and the number of on-body assays. As observed, there have been important advances in the field, but none of the reported devices reached the analytical and technological readiness needed for full and reliable application in the sports domain. Consequently, sweat lactate sensors have grown in popularity and importance in the scientific literature, while trustworthy commercial solutions remain in the early stages of development.

In this paper, we present a fully integrated concept for a wearable sweat lactate detection device, which is a clear step forward in demonstrating the significance of wearable sensors and sweat assessment in real-life scenarios. The concept’s design is based on the combination of a disposable electrochemical lactate biosensor, a microfluidic system for sample collection encompassing perspiration, an electronic board for wireless data acquisition and processing, and a user-friendly mobile application. On the one hand, the biosensor mechanism permits accurate and continuous lactate monitoring in sweat during the entire sports practice period. On the other hand, the reliability and robustness of the developed system allowed it to be used for sports physiology studies across different sports disciplines (cycling and kayaking), demonstrating great potential not only within the sports science domain but also as a possible commercial solution.

## Experimental Section

### Fully Integrated Wearable System for Sweat Lactate Detection

**Figure 1:**
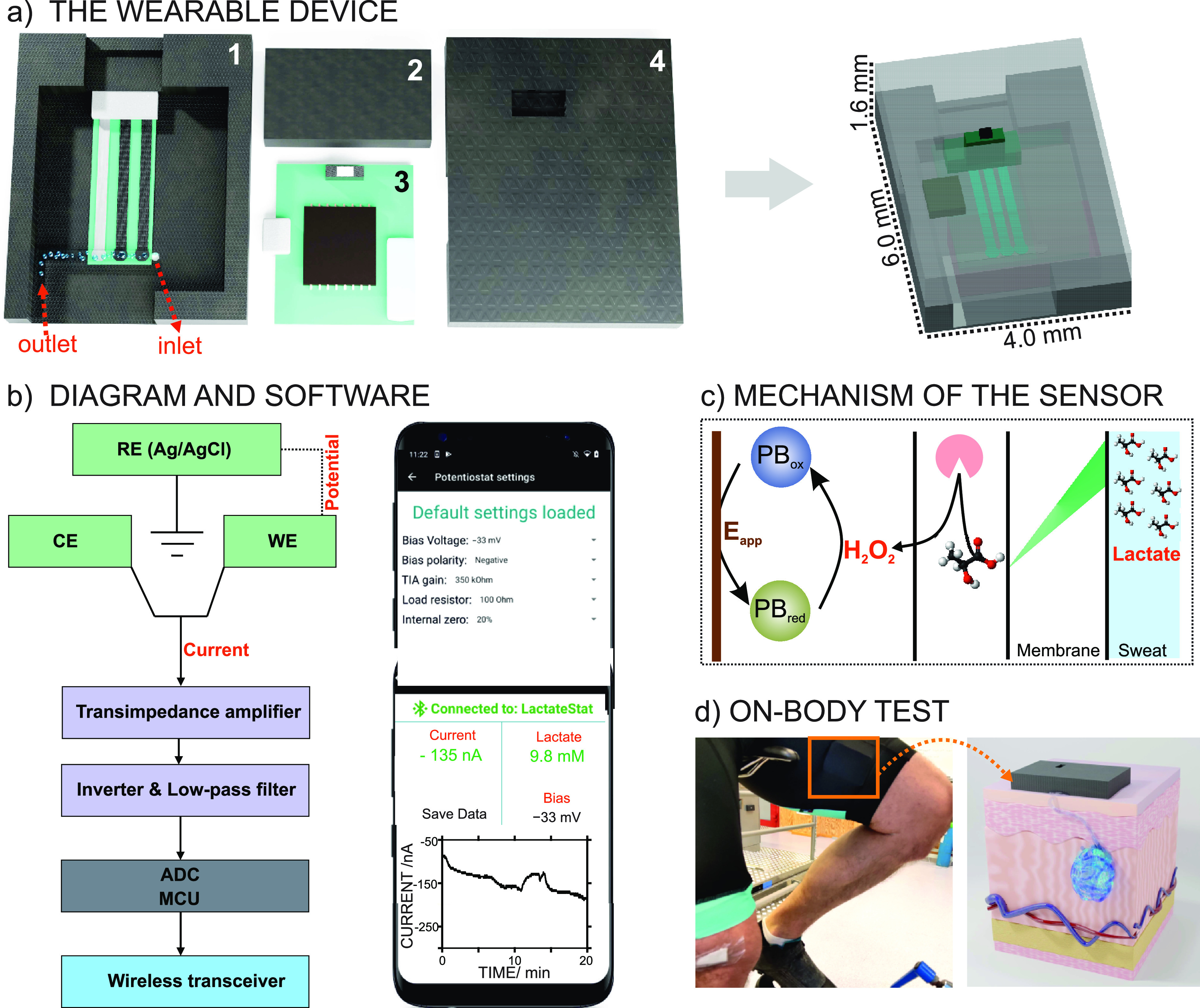
(a) Scheme of the wearable device: (1) microfluidic cell containing the lactate biosensor, with inlet and outlet for sweat flow; (2) pressure controller; (3) the custom electronics; and (4) upper container of the system. (b) Illustration of the system-level block diagram (left) and the control panel interface for adjusting the electronics and visualization of the real-time signal via a custom phone application (right). (c) Working mechanism underlying the lactate biosensor. (d) Photograph of an on-body test in which the wearable device is placed on the thigh of a cyclist. RE = reference electrode. CE = counter electrode. WE = working electrode. PB = Prussian blue. Ox = oxidized. Red = reduced. *E*_app_ = applied potential.

The system (Figure 1a) consists of (i) an enzyme-based electrochemical biosensor coupled to an outer diffusion-limiting membrane that allows sweat lactate determination, (ii) a 3D-printed sweat sampling cell and pressure controller (cuboid, 30 × 16 × 7 mm), (iii) a reusable electronic board for signal (current) recording and wireless transmission, and (iv) a custom mobile application. A photograph of each part is shown in Figure S1 in the Supporting Information.

The lactate biosensor comprises a three-electrode system (working, reference, and counter electrodes) manufactured in a flexible polymeric substrate. The electronic circuit (designed with KidCAD) applies a fixed potential of −0.033 V to the working electrode with respect to the reference (Ag/AgCl electrode) while measuring the current between the working and counter electrodes. An amperometry circuitry reads the electrochemical sensor, and the microcontroller unit (MCU) is required to initiate and communicate with the amperometry circuitry. A built-in Bluetooth low energy transceiver controlled by the MCU was used to communicate with the custom mobile application.

### Protocols for the On-Body Tests

All measurements were done in sports laboratories at an ambient temperature of 20 °C and 40% relative humidity. Parameters such as height, weight, gender, and the targeted body part were annotated before performing each test. These are provided in the Supporting Information (Table S2).

In the cycling tests at Dalarna University (Sweden), the heart rate, VO_2_, and carbon dioxide output (VCO_2_) were monitored through a metabolic chart in mixing chamber mode (Jaeger Oxycon Pro, Erich Jaeger GmbH, Hoechberg, Germany). The cycling intensity and power output were recorded using a cycle ergometer (LC7, Monark Exercise AB, Vansbro, Sweden). Before data collection, each participant performed a 9 min warm-up on the ergometer exercise bike. The warm-up consisted of cycling for 3 min at three different cycling intensities (1.2, 1.4, and 1.6 W per kg). Then, the training consisted of four 15 min rounds at different intensities, with ca. 2 min of rest between each round. Every 5 min, the subject was asked for a Borg scale number to describe their feeling of exhaustion (6 means “no exertion at all”, and 20 represents “maximal exertion”). Blood samples were collected during each resting period for lactate and glucose analysis. Sweat samples were also collected during the resting periods to validate the lactate measurements provided by the new device.

Max-power tests in cycling and kayaking were carried out at the Swedish Sport Confederation Performance Laboratory (Boson, Sweden). In this case, blood, sweat, and power output information were recorded. For cycling, a 20 min warm-up and a 20 min self-paced maximal sustainable effort were performed on a magnetic brake cycle ergometer (AtomX, Wattbike, UK). Blood samples were collected before and after the warm-up and after finishing the test. The kayaker performed a testing protocol consisting of paddling on four intensity levels (4 min for each level) and a final self-paced maximal sustainable power step (4 min) using a kayak ergometer (Dansprint, Hvidovre, Denmark). Blood samples were collected within 1 min after each step.

## Results and Discussion

### Wearable Device for On-Body Sweat Lactate Monitoring

The fully integrated wearable device for sweat lactate monitoring developed in this paper is presented in Figure 1. The three electrodes that constitute the sensing element are embedded in a microfluidic channel with an inlet and an outlet to create a sweat flow on the surface of the electrodes (Figure 1a). A pressure controller with a cuboid geometry was placed between the microfluidic channel and the upper case to minimize pressure changes occurring during physical activity. Overall, the design of the sweat sampling cell ensures that the sweat flow generated on the surface of the electrodes is almost the same as the perspiration rate in the subject.

The system-level block diagram of the device is given in Figure 1b, displaying the current signal obtained from the lactate biosensor in potentiostat-like electronics (green), the processing of the raw current signal (purple), the analog-to-digital converter (gray), and the wireless communication (blue) between the electronic board and the user interface on the mobile phone.

The biosensing element follows the “first-generation” concept and consists of three primary parts: the redox mediator, the enzyme, and the external diffusion-limiting layer. The working mechanism is illustrated in Figure 1c. Briefly, the external diffusion-limiting membrane controls lactate transport from the sample to the enzyme layer, maintaining a steady level. Then, the lactate interacts with the lactate oxidase enzyme, producing hydrogen peroxide and pyruvate. Prussian blue reduces the hydrogen peroxide, which can be monitored from the decreasing current, which is proportional to the amount of lactate in the sample. A more detailed description of the working mechanism is presented in the Supporting Information.

The device can be placed (via medical tape and straps) on any part of the body without causing discomfort to the subject while practicing sports. Figure 1d shows how the gadget can be attached to the skin, in this case, under the sweatpants of a cyclist. Once attached, the current signal is recorded and transferred via Bluetooth to the custom mobile application. When the subject’s perspiration reaches a certain level, the generated sweat flows through the microfluidic channel, passing over the detection zone, and the current acquired at that precise moment translates into a meaningful sweat lactate concentration.

### Performance of the Lactate Biosensor in Batch Mode

**Figure 2:**
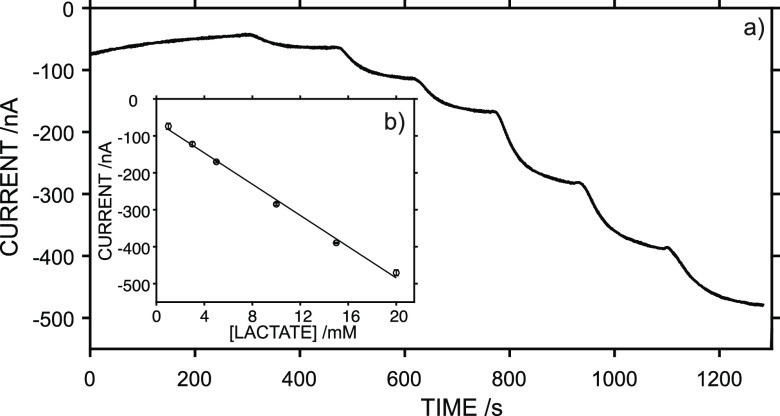
(a) Dynamic response of the lactate biosensor toward increasing lactate concentrations in artificial sweat. (b) Average calibration graph from three consecutive measurements using the same biosensor. Error bars refer to the standard deviation of the current responses for each concentration (n=3).

The electroanalytical performance of the lactate biosensor was evaluated in vitro in both batch and flow modes. For the batch mode, the experiments were accomplished in artificial sweat at a constant stirring rate of 150 rpm. Figure 2a shows a typical calibration graph obtained at lactate concentrations increasing from 1 to 20 mM. The average response time (defined as the time needed to achieve the 95% of the steady-state current response) was <80 s. The lactate response was repeatable when subsequent calibrations were performed with the same biosensor (Figure 2b). Variations of 0.6 and 7.2% were found for the average slope and intercept, respectively, from three consecutive calibration graphs. Overall, the linearity covered a concentration range of 1–20 mM, with a slope of −21.1 ± 0.57 nA mM^–1^, LOD of 0.12 mM, and intercept of −61.7 ± 7.43 nA. Importantly, the biosensor response included the typical concentration interval of sweat lactate.^25,26^

Sensor-to-sensor reproducibility was evaluated from the calibration graphs obtained with three identically prepared sensors (Figure S2 in the Supporting Information). The LRR was well-maintained (1–20 mM), as well as the slope (−21.5 ± 0.91 nA mM^–1^, 0.8% of variation) and LOD (0.2 mM). The biggest difference was observed in the intercept (−94.1 ± 33.97 nA, 34% of variation), which is intrinsic to the variability in the fabrication process. Accordingly, each biosensor will require pre-calibration for further on-body tests.

Interference was assessed by registering the response of the biosensor in 5 mM lactate and then adding 0.1 mM uric acid, 0.1 mM pyruvate, 0.1 mM ascorbic acid, or 0.25 mM glucose, which are above typical concentrations of these compounds in sweat (Figure S3). The lactate sensor showed a negligible response to these compounds, which makes it directly applicable to sweat lactate measurements without any potential interference. Moreover, the resilience of the biosensor was evaluated by inspecting the calibration curves obtained before and after bending the system 10 and 30 times to 45° (Figure S4). Conveniently, the slope and intercept were only slightly modified after this strong torsion strain, with RSDs of 2.7 and 12.0 after 10 and 30 bends, respectively. Accordingly, the biosensor response is not expected to be affected by subject movements during sports.

The effect of pH and temperature on the sensor performance was also investigated (Figure S5 in the Supporting Information). Four calibrations were conducted at 22 °C, with a successive decrease in the pH of the solution: 8.1, 6.9, 5.4, and 4.3. Then, another three calibrations were performed at a constant pH of 8.1 and varied temperatures: 22, 29, and 36.5 °C. The pH fluctuation caused an overall variation in the slope and sensitivity of ca. 8%, whereas for the temperature, the variations were 10 and 23% for the slope and intercept, respectively. On the one hand, the variation connected to pH changes is much lower than those previously reported for other enzyme-based sensors (e.g., ∼300% of variation for pH 4–8).^27,28^ This improvement is likely attributed to the external layer added to the lactate biosensor, which limits the number of protons reaching the enzyme from the sample, as suggested previously.^9^

The lifetime of the biosensor was assessed using a set of five similarly prepared sensors, calibrating each one several days after being prepared: 7, 12, 14, 18, and 29 days. The sensors were stored at 4 °C until they were tested. The five calibration graphs are presented in Figure S6 in the Supporting Information. No significant changes were observed in the slope (−22.0 ± 0.97 nA mM^–1^; RSD < 5%). However, the RSD for the intercept was >10%, which can be associated with the inherent variations between electrodes (see above). In any case, the response began to deteriorate 30 days after fabrication.

### Performance of the Lactate Biosensor in Flow Mode

**Figure 3:**
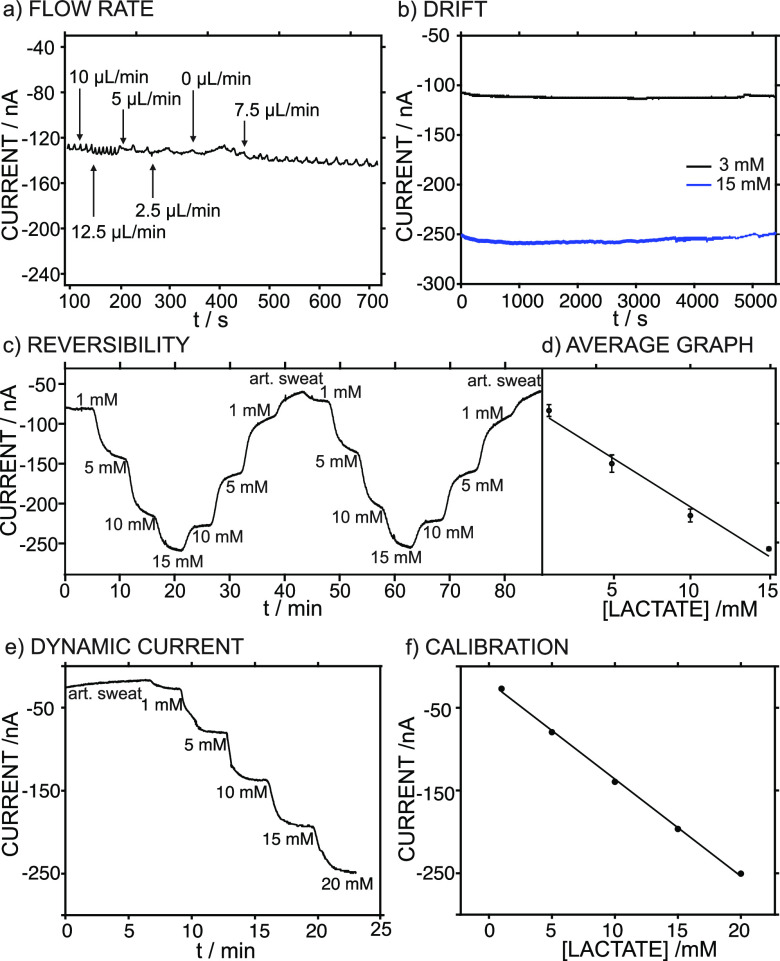
(a) Dynamic current for 5 mM lactate concentration at different flow rates in the device. (b) Medium-term drifts at 3 and 15 mM lactate. (c,d) Reversibility study: dynamic response and the corresponding average calibration graph. Error bars refer to the standard deviation of the current responses for each concentration (n=4).(e,f) Typical pre-calibration experiment performed prior to on-body tests: dynamic response and the corresponding calibration graph.

Considering that the goal of the lactate biosensor is a wearable device for continuous lactate monitoring, the sensor was implemented into the sampling cell to evaluate the performance under flow conditions. This was achieved by connecting a peristaltic pump to the inlet of the cell. Because the perspiration rate may vary during physical activity and differences between individuals are expected, the effect of the flow rate on the sensor response was investigated within a range that mimics common fluctuations in perspiration (from 2 to 7 μL cm^–2^ min^–1^).^29^ Flow rates adjusted from 0 to 12.5 μL min^–1^ emulate sweat rates from 0 to 13.7 μL cm^–2^ min^–1^ in the microfluidic channel when the area of the microfluidic channel (0.9 cm^2^) is considered. Thus, a solution containing 5 mM lactate was introduced to the cell through a peristaltic pump at 10 μL min^–1^, the flow was maintained, and the current was developed. Once the steady-state current was reached, the pump provided different flow rates, as shown in Figure 3a. The system response did not show any significant changes, highlighting its suitability for continuous sweat monitoring with respect to the perspiration of the subject.

The response stability was studied by obtaining the mid-term drift (defined as the current change within 1.5 h, mimicking the typical exercise period). Two lactate concentrations within the LRR were investigated, and drifts of 3.3 and 6 nA h^–1^ were observed for 3 and 15 mM lactate, respectively (Figure 3b). The reversibility of the response was characterized by consecutively increasing and decreasing the lactate concentration: the dynamic current was recorded during two complete cycles from 1 to 15 mM lactate and vice versa. The results are shown in Figure 3c and yield the average calibration curve in Figure 3d. The sensitivity was −12.5 ± 0.53 nA mM^–1^ and the intercept was −80.5 ± 10.7 nA, with variation coefficients of 4 and 13%, respectively. A lower sensitivity than that observed in batch mode was obtained, which could be attributed to the change in the mass transport regime of lactate to the sensing interface. All these analytical characteristics support the suitability of the developed biosensor for the on-body monitoring of sweat lactate, which is demonstrated in the next section.

Before each on-body test, the device was calibrated in flow mode using a peristaltic pump with a constant flow rate of 5 μL min^–1^ using artificial sweat as the background. A typical profile of the dynamic current at increasing lactate concentrations and the corresponding calibration graph are depicted in Figure 3e,f, respectively. Five concentrations of lactate (1, 5, 10, 15, and 20 mM) were selected, covering the levels expected for sweat lactate and providing a significant number of calibration points to reduce the probability of further error when sweat lactate is determined. Effectively, this calibration graph transforms raw current data profiles from on-body tests into dynamic sweat lactate concentrations.

### On-Body Tests at Dalarna University: Investigation of the Relationship between Sweat and Blood Lactate

**Figure 4:**
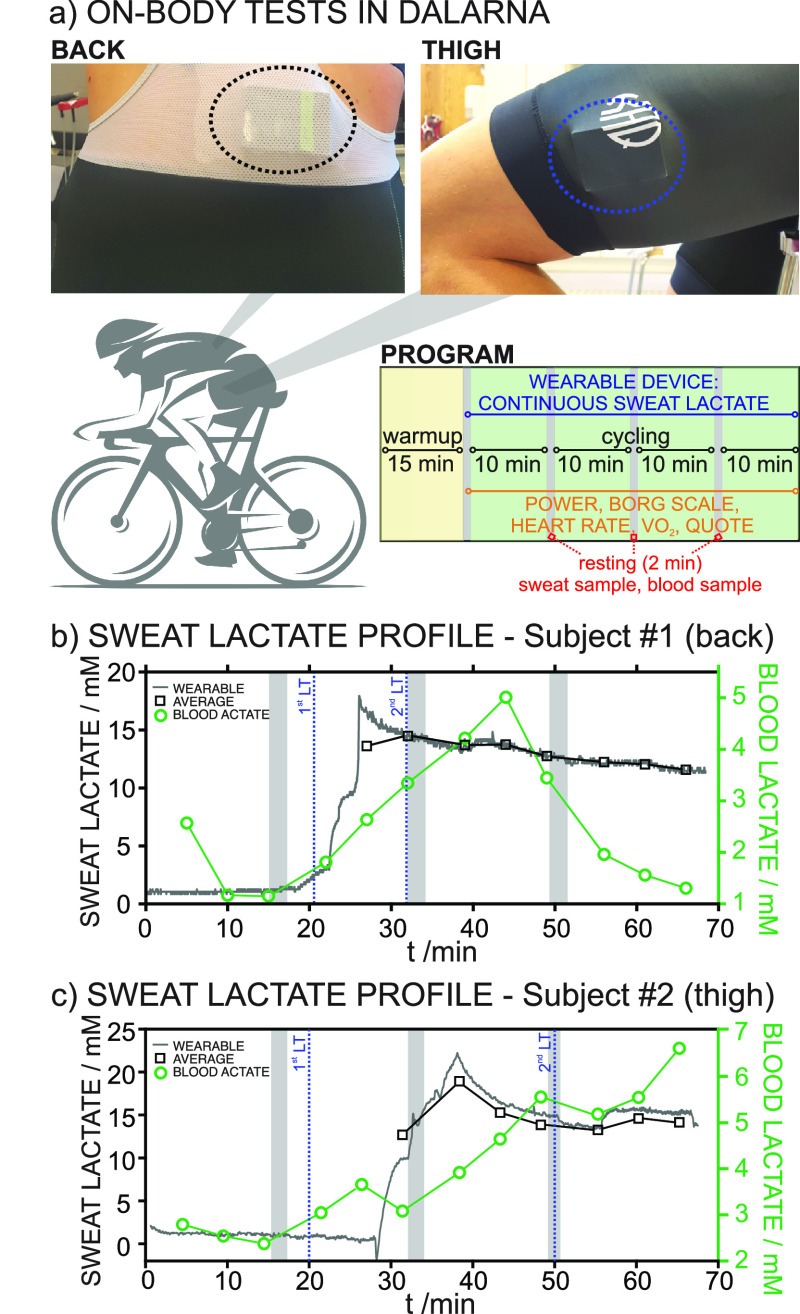
(a) Photos of on-body tests conducted at Dalarna University, with the devices positioned on the back and thigh, and schematics of the training protocol. (b,c) Profiles for the dynamic sweat lactate observed in the on-body tests with subject #1 (back) and subject #2 (thigh). Sweat lactate averaged every 5 min and blood lactate analyzed from samples collected every 5 min are also presented. Gray squares indicate the resting periods. Blue lines indicate the first and second lactate thresholds (LTs) determined from the blood lactate levels.

On-body tests were performed with the device positioned on two different body parts (Figure 4a): four subjects with the device on the back and five subjects with the device on the thigh (Table S2 in the Supporting Information). Each participant was required to cycle for ca. 76 min divided into 10 min of warm-up and four 15 min periods of different intensities (each period followed by 2 min of rest). In parallel to the sweat lactate measurements, sweat and blood samples were collected. Lactate was determined from all the samples, and glucose was only determined from the blood samples. Also, exhaustion level (RPE, Borg scale), power, heart rate, VO_2_, and respiratory exchange ratio were monitored.

Figure 4b,c depicts two of the dynamic sweat lactate profiles observed on the back and thigh of subject #1 and subject #2 and also include the sweat lactate (averaged every 5 min from the continuous measurements) and blood lactate calculated from the samples collected during the resting periods every 5 min. Notably, the back is a more passive area than the thigh when cycling. Also, the perspiration level on the back is higher than that on the thigh. In both subjects, there was an initial period of constant concentration followed by a sudden increase at 18 and 27 min, corresponding to the time needed for the device to reach the perspiration level of each subject at the specific body part, i.e., sufficient sweat reaching the electrodes. In essence, initial measurements are not reliable until this point. As a general trend, longer initial times were required for the sensor on the thigh than the one on the back due to the lower perspiration rate.

In subject #1 (Figure 4b), after the initial increase observed from 18 to 26 min, the sweat lactate measured on the back decreased during the rest of the practice. Conversely, blood lactate was constant for the first 20 min, then rose from 1.8 to 5.0 mM until 45 min had passed, and next decreased until a value close to the initial one was obtained (1.3–2.0 mM). The first and second LT occurred at ca. 20 and 32 min, with the final decrease of blood lactate coinciding with decreased training intensity. The two LTs delimit zones 1, 2, and 3 in the training practice according to the level of intensity, as shown in Figure S7 in the Supporting Information. The sweat lactate was 4.3–5.2 times higher than the blood lactate in zone 2, 2.7–3.7 times higher in zone 3, and 6.2–8.9 times higher in the decreasing blood lactate zone.

In subject #2 (Figure 4c), after the initial concentration increase observed from 28 to 38 min, the sweat lactate measured in the thigh decreased from 18.9 mM down to 14.8 mM until ca. 55 min (coinciding with zone 2 for blood lactate), later increasing to 16.0 mM and remaining constant afterward (15.8 ± 0.2 mM), coinciding with zone 3. There was an additional final decrease in parallel with decreased exercise intensity. The sweat lactate was 2.8–5.0 and 2.3–2.8 times higher than the blood lactate in zones 2 and 3, respectively.

Considering the lactate profiles observed in the remaining subjects (Figure S8), when the measurements were taken on the back (subjects #3–5), sweat lactate tended to be either almost constant or decreased when the blood lactate increased, coinciding with the trends shown for subject #1. After reaching the level of perspiration needed to obtain the measurements from the device, subject #3 presented a lower perspiration level than the rest. Because this individual showed almost constant sweat lactate, while the rest showed decreasing lactate, a hypothesis may be drawn. In principle, significant changes in sweat lactate may not be observed by targeting a relatively passive body zone, such as the back, during cycling. Decreasing sweat lactate coinciding with increasing activity intensity (and thus blood lactate) was not expected a priori. Thus, there may be a dilution of the real (and relatively constant) sweat lactate concentration caused by high perspiration levels in the individual. This effect has been suggested previously.^23^

**Figure 5:**
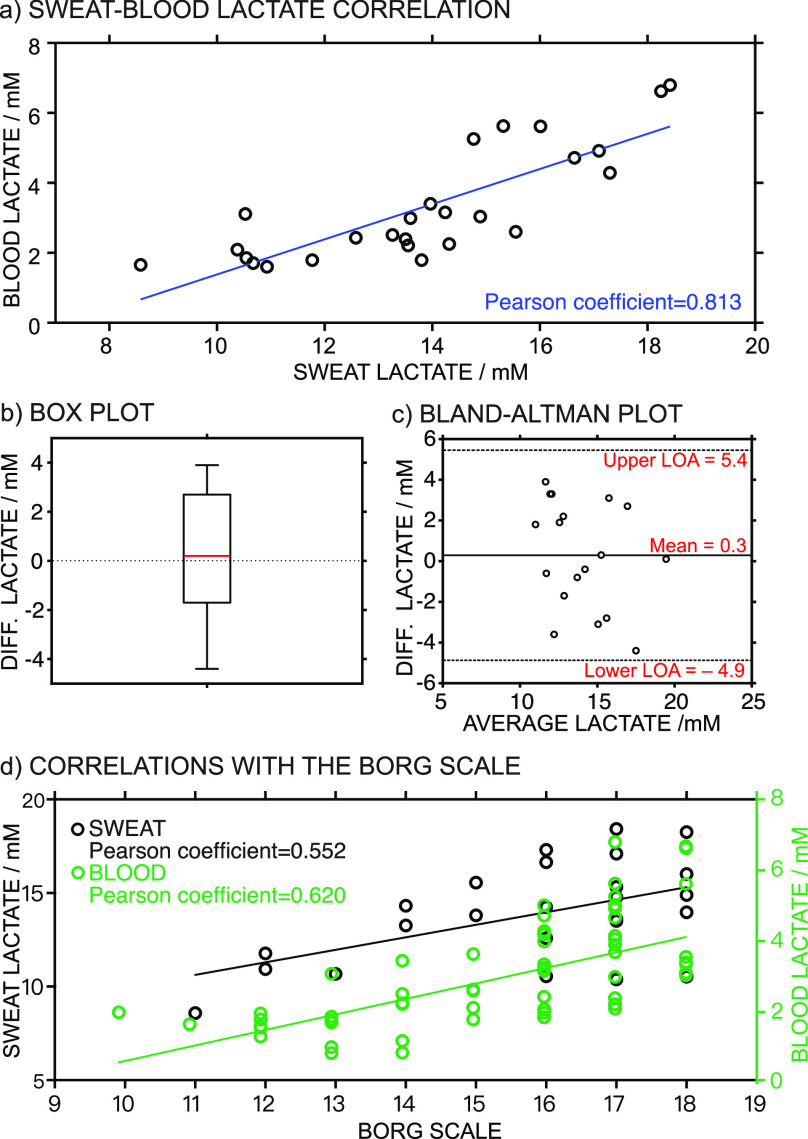
(a) Correlation between sweat lactate (on the thigh) and blood lactate. (b) Box plot of the validation of sweat lactate measurements. (c) Error distribution of the validation of sweat lactate measurements. (d) Correlation between sweat lactate and Borg scale, as well as blood lactate and Borg scale.

Regarding measurements on the thigh, a different situation was found. In general, times slightly longer than in the back were needed to initiate the measurements with the wearable device. There appeared to be a positive relationship between sweat and blood lactate because both increased and remained constant almost simultaneously with the blood lactate zones. Accordingly, we further inspected this data. As shown in Figure 5a, a correlation plot between sweat lactate (averaged every 5 min from the data provided by the wearable device) and blood lactate indicated a Pearson coefficient of 0.813 (27 samples, not considering those samples with sweat lactate ≥20 mM, which is higher than the LRR of the biosensor), confirming a positive correlation when a value of 0.80 was used as the cutoff.

More detailed descriptions and interpretations of the outcomes observed for each subject are provided in Section S4 in the Supporting Information. On-body measurements conducted on the thigh during cycling seemed more promising than those on the back. Hence, we further focused on these data. In any case, the sweat lactate measurements were validated, and the accuracy was confirmed (next section). Thus, all the conclusions drawn in this paper can be considered valid.

### Validation of Sweat Lactate Measurements Obtained with the Wearable Device

In parallel with the real-time, continuous, and on-body sweat lactate monitoring using the wearable device, sweat samples were collected every 5 min from a body position very close to the device. The lactate contents in the samples were analyzed by ion chromatography (IC) and then compared with the discrete values averaged over periods of 15 min (coinciding with the sampling) from the continuous data provided by the wearable device. Although the collected sweat volume was insufficient to be analyzed by IC in some cases, we assessed a total of 18 sweat samples. The results are shown in Table S3 in the Supporting Information.

Considering a cut-off value of 20% for the difference between both techniques, 80% of the samples agreed with the calculated sweat lactate, confirming the reliability of the measurements. Importantly, significant differences may arise from comparing continuous data to discrete profiles, and to address this, we had to use averaged results from the dynamic lactate profiles, which may induce some errors.

A dependent sample *t*-test was used to estimate whether the average difference between the results from the wearable device and IC is null considering a confidence interval of 95%. A value of the *t*-score (0.47) lower than the critical theoretical value (2.11) was obtained, indicating no statistical differences between the results obtained from the two analytical methods. The boxplot of the difference values (Figure 5b) presents a near-zero value calculated from most samples, with a median value of 0.2 mM, a first quartile value of −1.7 mM, and a third quartile value of 2.7 mM. The Bland–Altman plot (Figure 5c) was also applied for a closer inspection of the individual agreement between samples, identifying trends but also some inconsistencies in variability across the lactate concentration range (from 10 to 20 mM). The error distribution was homogeneous along the lactate concentration range of 11–19.5 mM, indicating that variability and discrepancy are not dependent on the observed lactate concentration.

### Investigation of the Relationship between Sweat Lactate (on the Thigh) and Other Physiological Parameters

The Borg scale, an indicator of the perceived exertion of the body, was selected to understand whether sweat lactate can be used as a proxy for sports performance. Borg scale grading commonly differs among subjects facing analogous physical activity owing to different body conditions. It is a measurement of how hard the subject feels the body working, and it is based on the physical sensations experienced during physical activity, which normally coincide with increasing heart rate, respiration (or breathing rate), perspiration, and muscle fatigue; this latter is connected to blood lactate.^30,31^ Indeed, perceived exhaustion is easily obtained from the subject without disturbing the sports activity. Accordingly, the data from each subject were grouped per the Borg scale (Table S4), and all the possible correlations were analyzed. Notably, while we only considered sweat lactate measurements from the thigh (subjects #2 and #3–6), all the data (i.e., the nine subjects) were used for the rest of the parameters.

Correlations found for the sweat and blood lactate with the Borg scale are presented in Figure 5d, while the other parameters (blood glucose, power, heart rate, VO_2_, and respiratory quotient) are shown in Figure S9 in the Supporting Information. Importantly, as expected, the perceived exhaustion positively correlated with the heart rate (cutoff of 0.6 for a subjective variable). This occurred for the entire pool of samples (*n* = 70) and when analyzing the data for each subject (Table S5). Indeed, the ratio between the heart rate and the Borg scale was always close to 10 (Table S6), confirming the reliability of the data.^32^ The respiratory quotient was also found to positively correlate with the Borg scale in connection with increasing anaerobic respiration and the main metabolism of proteins (values ranged from 0.8 to 0.95).^33^ Once more, this was observed for the entire pool of samples and each subject (Table S7). No clear correlations were found for power, blood glucose, or VO_2_ (Figure S9).

Regarding lactate, as shown in Figure 5c, both sweat lactate (measured on the thigh) and blood lactate showed positive correlations with the Borg scale (Pearson coefficients of ca. 0.6) and, thus, with the heart rate and the respiratory quotient. This may mean that an increased level of perceived exertion can be attributed to the increased lactate concentration in the sweat generated in the zone of the thigh, the active muscle, during cycling. Individual correlations for each subject also revealed positive correlations (Tables S8 and S9).

In summary, we found promising positive correlations between the sweat lactate measured on the thigh with blood lactate, perceived exertion, heart rate, and the respiratory quotient. The device developed in this study can be used to precisely measure sweat lactate and relate this to personalized sports performance via the perceived exertion level and the heart rate, two parameters that are easy to measure, accessible to professionals and amateurs, and that can be incorporated into the software of the device developed in this paper. However, we believe that back measurements could also be targeted upon dilution correction based on additional measurements with a sweat rate sensor.

### Tests for Cyclists and Kayakers at the Swedish Sport Confederation Performance Laboratory (Bosön)

**Figure 6:**
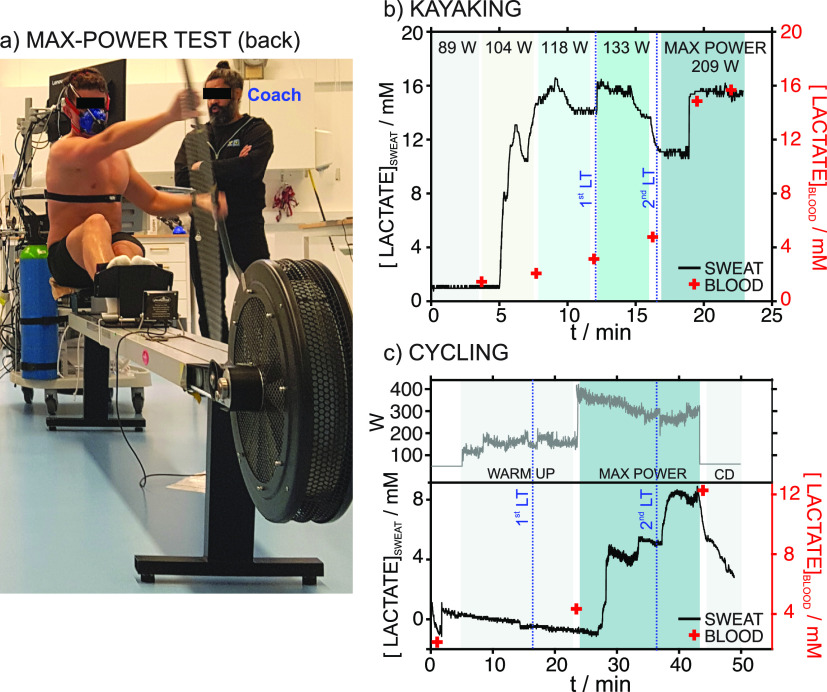
(a) Image of maximal power test in kayaking with the wearable sensor placed on the back of the participant. (b) Dynamic profile for sweat lactate obtained from the back of the kayaker in the maximal power test. (c) Dynamic profile for sweat lactate obtained from the back of the cyclist in the maximal power test. Blood lactate measurements are also included with the LT (blue lines, LT). The power of the exercise is indicated for reference. CD = cool down.

To demonstrate the versatility of the developed system, we explored its use in elite kayakers and cyclists during submaximal and maximal self-paced tests. In kayaking, the upper body musculature plays a critical role, in contrast to cycling, where lower limb musculature consists of the propulsive muscles. Figure 6a shows a picture of a test setup in a kayaker wearing the device on the upper back. In addition to the dynamic sweat lactate profile recorded during the entire test, blood lactate measurements (finger-prick method) and power output were assessed. The results are shown in Figure 6b for the kayaker and Figure 6c for the cyclist.

The subject of the kayaking test accomplished four intensity steps, increasing in power (89–133 watts), followed by 1 min rest periods, before performing the maximal power period. The device started the lactate measurements (i.e., sufficient sweat reached the electrodes) after ca. 5 min. In the warm-up, a clear increase of sweat lactate up to 16.7 mM was observed from 5 to 10 min, followed by a gradual decrease to 14.3 mM, coinciding with the middle part of the 118 watt-step. This concentration was constant until the end of the step. Then, a similar trend was found in the 133 watt-step: an initial increase to 16.4 mM, maintained until the middle of the step, a gradual decrease to 13.8 mM, and maintenance of this concentration until the end of the warm-up. Before the maximal power step, the sweat lactate decreased to 11.2 mM, suddenly increased to 15.7 mM after some minutes, and remained constant. Interestingly, this latter trend coincided with the second blood LT. Advantageously, the device was able to monitor changes in the sweat lactate that may be related to the performance of the active muscles in the measured body area.

The subject of the cycling test accomplished a free warm-up (power between 120 and 180 watts), 20 min of maximal power exercise (intensity ranging from 229 to 400 watts), and a final cool-down period (60 watts for 5 min). A free warm-up protocol in which the participants mainly controlled the workout power was used. The device started the lactate measurements from ca. 28 min, longer than for the kayaker because kayaking is a whole-body exercise, and thus, more sweating is expected. Hence, the lactate was monitored only during the 20 min self-paced maximal sustainable cycling step. The device detected an initial increase in sweat lactate to 4.5 mM, which was constant for 4.5 min before increasing again to 5.4 mM. This concentration was constant for some minutes before rising to 8.4 mM; then, it remained constant until the end of the maximal power activity. This last increase seems to coincide with the second LT. During the calm-down period, sweat lactate was found to gradually decrease until it reached basal levels.

On comparing both activities, cycling revealed three lactate increases during the maximal power step in contrast to only one in kayaking. In any case, the final increase coincided with the second blood LT. Both sweat and blood lactate levels were higher in the kayaker than in the cyclist. For sweat lactate, this could be caused by using a more active muscle area in the kayaker than in the cyclist. Overall, the device provided excellent performance in both cases.

## Conclusions

Wearable sensors to digitize sweat lactate are positioned as a feasible alternative to traditional blood test protocols. The device herein developed demonstrated not only this but also the significance of sweat lactate in sports performance monitoring. Advantageously, our fully integrated system combines an exhaustive analytical characterization and rigorous accuracy with outcomes from different on-body cases. The device features an advanced microfluidics design for efficient sweat collection and analysis, lactate biosensors that reversibly cover the expected range for sweat lactate levels with a relatively fast response time, an integrated electronic circuit for signal processing, and a custom smartphone application. The lactate biosensor response is negligibly influenced by changes in pH, temperature, flow rate, and certain chemical interferences. Importantly, in cycling, the device provided more engaging results on the thigh than on the back: this shows the difference between active and passive muscles. We found a positive correlation between the sweat lactate measured from the thigh and blood lactate, perceived exhaustion (measured with the Borg scale), heart rate, and the respiratory quotient. Moreover, the lactate measurement device is also suitable for monitoring maximal power kayaking and cycling. In terms of commercialization, challenging aspects such as lifetime, sweat rate correction, and physiological protocols focused on sweat lactate rather than blood may be tackled.

## Supporting Information Available

Analytical assessment, materials, and the device photo (PDF)
