# Supplementary material for: Fully Integrated Wearable Device for Continuous Sweat Lactate Monitoring in Sports
Source: ACS Sens. 2023 Jun 8;8(6):2401–9. doi: 10.1021/acssensors.3c00708 (PMC10294257; doi:10.1021/acssensors.3c00708)
Supplement: Supplementary file 1 — se3c00708_si_001.pdf [file se3c00708_si_001.pdf]

Supporting Information for:

Fully Integrated Wearable Device for Continuous Sweat Lactate Monitoring in Sports

Xing Xuan[1,2], Chen Chen[1], Agueda Molinero-Fernandez[1,2], Emil Ekelund[1], Daniele Cardinale[3], Mikael Swarén[4], Lars Wedholm[5], Maria Cuartero[1,2,*] and Gaston A. Crespo[1,2,*]

[1]*Department of Chemistry, KTH Royal Institute of Technology, Teknikringen 30, SE-100 44, Stockholm, Sweden.*
[2]*UCAM-SENS, Universidad Católica San Antonio de Murcia, UCAM HiTech, Avda. Andres Hernandez Ros 1, 30107, Murcia, Spain.*
[3]*Department of Physiology, Nutrition and Biomechanics, The Swedish School of Sport and Health Sciences, GIH, SE-11486, Stockholm, Sweden.*
[4]*Swedish Unit of Metrology in Sports, Institution of Health and Welfare, Dalarna University, SE-791 88 Falun, Sweden.*
[5]*Institution of Health and Welfare, Dalarna University, SE-791 88 Falun, Sweden.*

**Corresponding authors: Maria Cuartero (mariacb@kth.se); Gaston A. Crespo (gacp@kth.se)*

Table of Contents

1. Experimental Section **3**

Reagents, materials, and instrumentation. 3

Preparation of the fully integrated sensing system for sweat lactate determination. 3

Characterization of the lactate biosensor and the fully integrated device. 4

Sensing mechanism of the lactate biosensor. 5

Participants in the on-body tests. 5

Protocol for blood sample collection and analysis. 5

Protocol for sweat sample collection and analysis. 5

2. Tables **7**

Table S1. Summary of the characteristics of some wearable electrochemical lactate sensors 7

Table S2. Volunteers' anthropometric characteristics, gender, and body place for data collection 7

Table S3. Validation of on-body sweat lactate measurements 8

Table S4. For subjects #1–#9, the correspondence of collected data with the rating of perceived exertion 9

Table S5. Correlations between the Borg scale and heart rate for each subject. 10

Table S6. The ratio between heart rate and the Borg scale for each subject. 11

Table S7. Correlations between the Borg scale and the respiratory quote for each subject. 12

Table S8. Correlations between the Borg scale and the sweat lactate for each subject. 13

Table S9. Correlations between the Borg scale and the blood lactate for each subject. 14

3. Figures **15**

Figure S1. Pictures of (a) lactate sensor, (b) sampling cell, (c) electronics, and (d) software. 15

Figure S2. Average calibration curve observed in the reproducibility study (three twin sensors). 15

Figure S3. Interferences' study 16

Figure S4. Resiliency test 16

Figure S5. Dynamic calibrations observed at varying pH and T conditions 17

Figure S6. Lifetime investigation 17

Figure S7. Schematics of the differentiation of zones 1, 2 and 3 in the training depending on the blood lactate thresholds 18

Figure S8. Real-time sweat lactate profiles measured during on-body tests in Dalarna with the wearable device attached to the back or thigh 18

Figure S9. Correlations for the (a) heart rate, (b) power, (c) blood glucose, (d) VO_2, and (e) respiratory quotient with Borg scale 19

4. Outcomes and interpretations of the on-body tests regarding a possible relationship between sweat and blood lactate **20**

5. References **22**

1. Experimental Section

Reagents, materials, and instrumentation.

Potassium hexacyanoferrate(III) (CAS-13746-66-2, >98% purity), iron (III) chloride (CAS-7705-08-0, >97% purity), sodium l-lactate (CAS-867-56-1), chitosan (CAS-9012-76-4), Nafion® perfluorinated resin solution 5% (CAS-31175-20-9), bis(2-ethylhexyl)sebacate (DOS) (CAS-122-62-3, ≤97% purity), polyurethane Tecoflex SG80A (PU) (CAS- 68400-67-9), high molecular weight poly(vinyl chloride) (PVC), tetradodecylammonium tetrakis(4-chlorophenyl)borate (ETH 500), and tetrahydrofuran (THF) (CAS-109-99-9) were purchased from Sigma-Aldrich. Analytical grade chloride salts of ammonium (CAS-12125-02-9), magnesium (CAS-7786-30-3), potassium (CAS-7447-40-7), sodium (CAS-7647-14-5), as well as sodium carbonate (CAS-497-19-8) Hydrochlorid acid solution (CAS-7647-01-0), and sodium phosphate (CAS-7558-79-4) were also purchased from Sigma-Aldrich. Lactate oxidase (LOx) was purchased from Sorachim SA, Switzerland (LCO-301). Silver/silver chloride (Ag/AgCl) ink and carbon ink were purchased from Henkel, German.

All solutions were prepared in 18.2 $M\Omega^{-1}$ doubly deionized water (Milli-Q water systems, Merck Millipore). Artificial sweat containing 60 mM NaCl, 6 mM KCl, 5 mM NH_4Cl, 0.08 mM $MgCl_2$, 2.6 mM $NaHCO_3$ and 0.04 mM Na_2HPO_4 was used as the background in all the experiments for the biosensor characterization.

PU filament for the 3D printing of the sampling cell, TPU 95A, Ultimaker Material 1756 was purchased from Ultimaker B.V., Netherlands. Electronics for signal (current) recording was designed through KiCAD, and manufactured at PCBWay, China. Polyester sheets (thickness of 100 μm) to fabricate screen-printed carbon and Ag/AgCl electrode paths (for further modification to provide the lactate biosensor) were purchased from RS components (Sweden). Macroduct device for sweat collection was purchased from ELITechGroup (The Netherlands).

Preparation of the fully integrated sensing system for sweat lactate determination.

The mask for screen-printing carbon and Ag/AgCl inks contained circular (diameter of 1.5 mm, sensing area) and rectangular (width = 1 mm, length = 20 mm, for the connection with electronic) parts, designed and fabricated using a Silhouette Cameo cutter, Silhouette Inc, The Netherlands. Either carbon or Ag/AgCl inks were screen-printed through the mask onto the flexible substrate (polyester sheet, 4 x 23 mm) and dried in the oven at 100 °C for 1 h to form a conducting path for further modification.

The working electrode was prepared using a layer-by-layer modification method: a mediator layer of Prussian Blue (PB), a reaction layer of the lactate oxidase enzyme (LOx) entrapped in Nafion, and external diffusion-limiting membrane (containing PVC, plasticizer, and ETH500). First, the PB layer was electrodeposited on the circle part of the carbon electrode by cyclic voltammetry (from –0.5 V to 0.6 V, two cycles at a scan rate of 50 mV s^{-1}) in an aqueous solution containing 2.5 mM of $K_3Fe(CN)_6$ and $FeCl_3$, 100 mM KCl, and 100 mM HCl. Afterwards, the electrode was rinsed with 10 mM HCl, and annealed in the oven at 100 °C for 1 h. The enzyme layer was prepared by drop-casting 2.5 μL of a solution containing 15 mg/mL of LOx, 5 mg/mL BSA, and 0.5 wt% Nafion in 10 mM phosphate buffer solution. This layer was dried for 20 min at room temperature before the outer polymeric membrane was deposited. For the latter, 1.5 μL of a solution containing 3 mg/mL ETH 500, 33 mg/mL PVC,

and 66 mg/mL DOS in THF was drop-casted and dried at room temperature for 20 min. This results in a membrane with a thickness of about 10 μm (measured with a physical contact profilometer, Dektak 150 Stylus surface profiler, Veeco, USA). Finally, the working electrode was overnight conditioned in the artificial sweat while storage in the fridge (4 °C) before usage. The counter and reference electrodes were fabricated as reported elsewhere.[1]

The sweat sampling element consisted of a microfluidic cell, a pressure controller (cuboid, 30 x 16 x 7mm), and an upper container for electronics, which were designed and manufactured by AutoCAD and a 3D printer (Ultimaker B.V., The Netherlands). The sensors were then fixed in the microfluidic channel with a double adhesive tape to form the sweat channel. The width, length, and depth of the channel are 3.0, 30.0, and 0.3 mm, respectively. The lactate sensor was located close to the inlet, lining up with the microfluidic channel, to reduce the time required for perspiration to reach the sensing area. Afterwards, the pressure controller was inserted between the microfluidic channel and the upper case to lessen pressure changes brought on by physical activity. It helps to maintain a constant pressure between the sweat duct and inlet of the device. A commercial 3-pin connector (5-520315-3-ND, Digi-Key) attached to the electronic board was used to connect the three electrodes.

The electronic circuit applied a fixed potential between the working and reference electrodes while measuring the current between the working and counter electrodes and was designed by KidCAD. Briefly, an amperometry circuitry reads the electrochemical sensor and the micro control unit (MCU) was required to initiate and communicate with the amperometry circuitry. An inbuilt Bluetooth low energy (BLE) transceiver controlled by the MCU was used to communicate with the custom-developed mobile application. A rechargeable lithium polymer battery (3.7 V) with a voltage regulator was used to supply the proper operating voltage level. To integrate all the parts together, a general printed circuit board (PCB) was manufactured by assembling all the components on it. The final board had a size of 58 x 55 x 13 mm, the current consumption was only 7.8 mA, and the current measurement resolution was 0.5 nA. The LOD was 0.45 nA with the current measurement range of ± 750 μA.

Characterization of the lactate biosensor and the fully integrated device.

The electrochemical performance of the lactate biosensor was characterized in artificial sweat solution at room temperature with an applied constant potential of -0.33 V (vs. the Ag/AgCl element). Calibration curves in the batch mode were performed by standard lactate additions while stirring the solution with a magnetic bar at 200 rpm. Furthermore, the selectivity of the lactate biosensor was evaluated in artificial sweat containing potential interferences in human sweat, such as 250 μM glucose, 100 μM ascorbic acid (AA), 100 μM pyruvate and 100 μM uric acid (UA). The influence of temperature and pH was evaluated by performing calibration curves in artificial sweat at different pH and temperature conditions. Additionally, the performance of the device was evaluated in flow-mode using a peristaltic pump (ISMATEC IPC series, Cole-Parmer GmbH, Germany) to mimic perspiration. The influence of flow rate was tested at 10 μL /mL, 12.5 μL /mL, 5 μL /mL 2.5 μL /mL, 0 μL /mL, 7.5 μL /mL by controlling the pump.

Sensing mechanism of the lactate biosensor.

The sensing mechanism underlaying the lactate biosensor is based on the conversion of lactate at the enzyme layer, which produces hydrogen peroxide (H_2O_2) during enzymatic reaction. Importantly, the amount of lactate reaching the enzyme layer is controlled by the outer PVC membrane (containing ETH500). The resulting H_2O_2 is then detected through the mediator layer (mainly formed by PB_{red} at the applied potential), which reduces H_2O_2 spontaneously. The analytical signal (current) is built up by applying a cathodic potential to reduce back the fraction of the PB_{ox} formed due to the presence of H_2O_2. This fraction is proportional to the amount of H_2O_2 present in the system. The reactions can be summarized as follows:

$$Lactate + Lox + O_2 \rightleftarrows Pyruvate + H_2O_2 \quad (S1)$$

$$PB_{red} + H_2O_2 \rightleftarrows 2OH^- + PB_{ox} \quad (S2)$$

$$2e^- + 1/2PB_{ox} \rightleftarrows 1/2PB_{red} \quad (S3)$$

Participants in the on-body tests.

General on-body tests with thirteen cyclists and max-power tests with four athletes (two cyclists and two kayakers) were performed at Dalarna University and Boson Sports Center, respectively. Participants were asked not to use rosin before data collection to avoid contamination of the sweat samples. To avoid any disruption of data collection, all the participants were noticed to arrive in a rested and hydrated state before each on-body measurement. A total of eleven subjects were successfully monitored due to several technical reasons (e.g., low perspiration, connection issue between software and hardware, errors during data processing in software).

All the subjects provided written consent prior to the test. Ethical approval for this study was granted by the Swedish Ethical Review Authority (registration number: #2020-04206). All the research was conducted in accordance with the Code of Ethics of the World Medical Association (Declaration of Helsinki).

Protocol for blood sample collection and analysis.

20 µL of capillary blood from a fingertip were collected. The blood samples were immediately transported into a vial containing a standard solution from EKF-diagnostic GmbH (Barleben, Germany). The vials were subjected to shaking conditions for 20 min at a low speed using an automatic wiggle shaker (MiniMix, Molek AB, Årsta, Sweden) before measuring concentrations of blood lactate and glucose through BIOSEN C-line (EKF-diagnostic GmbH, Barleben, Germany).

Protocol for sweat sample collection and analysis.

Cotton pads for sweat collection were prepared by attaching a piece of cotton to a Hydrofilm water-resistant tape (Hartmann GmbH, Germany). The cotton pieces were previously rinsed with double distilled water and dried in the oven at 80 °C for 2 h. Before each round in the training program, the pads were positioned close to the lactate sensing device, and then promptly removed and squeezed using a syringe to extract the sweat. Sweat samples were stored in the fridge until further analysis. 5-mL syringes (HenkeSass Wolf, Germany) were used to squeeze the cotton pads to extract the sweat samples into an Eppendorf vial When

the Macroduct sweat collector was used, sweat samples were collected to an Eppendorf tube using a 1-mL syringe with the corresponding needle (HenkeSass Wolf, Germany). The sweat samples were packaged inside an ice bag, which can maintain the temperature under 10 °C to avoid any damage of the samples during transportation. The samples were then stored in a refrigerator at 4°C until analysis. The lactate contents in the collected sweat samples were analyzed in the laboratory using the IC (850 Professional IC, Metrohom, Switzerland): MetrosepA Supp 5–150/4.0 (ref. 6.1006.520) column, 1 mM $NaHCO_3$/3.2 mM Na_2CO_3 buffer as the mobile phase, flowrate of 0.8 mL min1 and 100 mM H_2SO_4 as the solution in the suppressor.

2. Tables

Table S1. Summary of the characteristics of some wearable electrochemical lactate sensors reported until the time of writing.

Validated?	Number of on-body tests	Readout system?	Microfluidics?	Physiological evaluation?	Slope (nA/mM)	LOD (mM)	LRR (mM)	Ref
No	2	No	No	No	644.2	0.2	0 – 20	[2]
No	0	No	Yes	No	30	–	0 – 10	[3]
No	4	Yes	No	No	200	–	5 – 25	[4]
No	3	Yes	Yes	No	102	0.35	0 – 15	[5]
No	6	No	No	No	4500	0.0002	1 – 100	[6]
No	2	Yes	Yes	No	490	0.003	0.3 – 20	[7]
No	3	Yes	No	No	1900	0.0008	0.2 – 25	[8]
Yes	5	Yes	Yes	No	2700	0.2	0 – 7	[9]
Yes	11	Yes	Yes	Yes	22	0.2	1 – 20	This work

Table S2. Volunteers' anthropometric characteristics, gender, and body place for data collection with the wearable lactate sensor.

Subject	Height (cm)	Body weight (kg)	Gender	Place for lactate measurements	Accomplished Institution
#1	176	76.4	Male	Back	Dalarna
#2	180	74	Male	Thigh	Dalarna
#3	182	86	Male	Back	Dalarna
#4	183	85.7	Male	Back	Dalarna
#5	187	85	Male	Back	Dalarna
#6	167	60	Male	Thigh	Dalarna
#7	180	73.3	Male	Thigh	Dalarna
#8	182	86	Male	Thigh	Dalarna
#9	170	57.9	Female	Thigh	Dalarna
#MC-1	175	74	Male	Back	Boson
#MK-1	174	72	Male	Back	Boson

MC: max-power cycling. MK: max-power kayaking.

Table S3. Validation of on-body sweat lactate measurements observed with the wearable biosensor. The results are compared with lactate concentrations in sweat samples collected every 15 min and analyzed with ion chromatography (IC) as the gold standard technique.

**Subject**	**Collection (min)**	**Body part**	**Lactate (mM)**		**Difference**
			**Biosensor**	**IC**	**Biosensor –IC (%)**
**#1**	17 – 32	Back	13.6	9.7	28.7
	34 – 49	Back	13.6	10.3	24.3
	51 – 66	Back	11.9	10.1	15.1
**#2**	17 – 32	Thigh	14.2	17.0	-19.7
**#3**	17 – 32	Back	11.4	12.0	-5.3
	34 – 49	Back	13.3	14.1	-6.0
	51 – 66	Back	13.7	10.4	24.1
**#4**	34 – 49	Back	15.3	19.7	-28.8
**#5**	17 – 32	Back	12.0	13.7	-14.2
	34 – 49	Back	15.4	15.1	1.9
	51 – 66	Back	13.9	11.7	15.8
**#6**	34 – 49	Thigh	17.3	14.2	17.9
	51 – 66	Thigh	18.3	15.6	14.8
**#7**	34 – 49	Thigh	19.5	19.4	0.5
**#8**	17 – 32	Thigh	14.0	14.4	-2.9
	51 – 66	Thigh	13.5	16.6	-23.0
**#9**	34 – 49	Thigh	10.4	14.0	-34.6
	51 – 66	Thigh	13.5	11.6	14.1

Table S4. For subjects #1–#9, the correspondence of collected data with the rating of perceived exertion (expressed in the Borg scale) measured every 5 min. Number of points = 70.

Subject	Body part	Number of data for each grade	Value in the Borg scale
**#1**	Back	3	12
		1	14
		1	15
		3	16
**#2**	Thigh	3	16
		2	17
		2	18
**#3**	Back	1	10
		1	12
		1	13
		1	15
		2	16
		3	17
**#4**	Back	2	13
		1	14
		1	15
		1	16
		4	17
**#5**	Back	2	13
		2	14
		2	15
		3	16
		1	17
		2	18
**#6**	Thigh	1	16
		2	17
		1	18
**#7**	Thigh	1	11
		1	12
		1	13
		2	14
		1	15
		1	16
		2	17
**#8**	Thigh	1	12
		1	13
		1	15
		2	16
		3	17
**#9**	Thigh	1	17
		3	18

Table S5. Correlations between the Borg scale and heart rate for each subject.

Subject	Body place	# of samples	Slope	Pearson coefficient
**#1**	Back	8	2.6	0.794
**#2**	Thigh	7	2.7	0.573
**#3**	Back	9	3.3	0.852
**#4**	Back	9	5.1	0.895
**#5**	Back	12	7.8	0.958
**#6**	Thigh	4	5.0	0.985
**#7**	Thigh	9	6.1	0.948
**#8**	Thigh	8	3.8	0.826
**#9**	Thigh	4	5.3	0.711

Table S6. The ratio between heart rate and the Borg scale for each subject.

Subjects	Borg scale	HR	HR/Borg
**#1 (back)**	15	170.00	11.3
	16	175.00	10.9
	16	176.00	11.0
	16	180.00	11.3
	14	168.00	12.0
	12	164.00	13.7
	12	166.00	13.8
	12	168.00	14.0
**#3 (back)**	10	144	14.4
	12	147	12.3
	13	151	11.6
	15	155	10.3
	16	157	9.8
	16	157	9.8
	17	166	9.8
	17	165	9.7
	17	171	10.1
**#4 (back)**	15	144	9.6
	16	142	8.9
	17	146	8.6
	17	149	8.8
	17	152	8.9
	17	152	8.9
	13	129	9.9
	14	130	9.3
	13	131	10.1
**#5 (back)**	13	135	10.4
	13	140	10.8
	14	144	10.3
	14	145	10.4
	15	149	9.9
	15	151	10.1
	16	155	9.7
	16	162	10.1
	16	164	10.3
	17	170	10.0
	18	173	9.6
	18	178	9.9
**#2 (thigh)**	16	148	9.3
	16	151	9.4
	16	153	9.6
	17	151	8.9
	17	150	8.8
	18	156	8.7
	18	157	8.7
**#6 (thigh)**	16	176	11.0
	17	181	10.6
	17	182	10.7
	18	186	10.3
**#7 (thigh)**	11	125	11.4
	12	126	10.5
	13	128	9.8
	14	139	9.9
	14	141	10.1
	15	142	9.5
	16	152	9.5
	17	156	9.2
	17	161	9.5
**#8 (thigh)**	12	147	12.3
	13	151	11.6
	15	155	10.3
	16	157	9.8
	16	157	9.8
	17	166	9.8
	17	165	9.7
	17	171	10.1
**#9 (thigh)**	17	176	10.4
	18	179	9.9
	18	182	10.1
	18	183	10.2

Table S7. Correlations between the Borg scale and the respiratory quote for each subject.

Subjects	Borg scale	RQ	Pearson Coefficient
**#1 (back)**	15	0.86	
	16	0.86	
	16	0.87	
	16	0.9	
	14	0.81	
	12	0.8	
	12	0.79	
	12	0.81	0.916
**#3 (back)**	10	0.85	
	12	0.84	
	13	0.85	
	15	0.87	
	16	0.86	
	16	0.84	
	17	0.85	
	17	0.86	
	17	0.85	0.276
**#4 (back)**	15	0.94	
	16	0.91	
	17	0.94	
	17	0.96	
	17	0.95	
	17	0.93	
	13	0.83	
	14	0.85	
	13	0.85	0.915
**#5 (back)**	13	0.86	
	13	0.89	
	14	0.88	
	14	0.88	
	15	0.88	
	15	0.86	
	16	0.87	
	16	0.88	
	16	0.87	
	17	0.9	
	18	0.91	
	18	0.89	0.544
**#2 (thigh)**	16	0.86	
	16	0.88	
	16	0.87	
	17	0.9	
	17	0.91	
	18	0.9	
	18	0.91	0.834
**#6 (thigh)**	16	0.9	
	17	0.92	
	17	0.94	
	18	0.94	0.853
**#7 (thigh)**	11	0.89	
	12	0.89	
	13	0.87	
	14	0.91	
	14	0.89	
	15	0.88	
	16	0.92	
	17	0.92	
	17	0.91	0.648
**#8 (thigh)**	12	0.84	
	13	0.85	
	15	0.87	
	16	0.86	
	16	0.84	
	17	0.85	
	17	0.86	
	17	0.85	0.305
**#9 (thigh)**	17	0.91	
	18	0.93	
	18	0.92	
	18	0.92	0.816

Table S8. Correlations between the Borg scale and the sweat lactate for each subject.

Subjects	Borg scale	Sweat Lactate / mM	Pearson Coefficient
**#1 (back)**	15	13.6	
	16	14.5	
	16	13.7	
	16	13.7	
	14	12.7	
	12	12.2	
	12	12.0	
	12	11.6	0.948
**#3 (back)**	10	9.2	
	12	12.0	
	13	12.4	
	15	12.2	
	16	13.8	
	16	13.9	
	17	13.0	
	17	14.0	
	17	14.2	0.904
**#4 (back)**	15	13.6	
	16	19.4	
	17	17.6	
	17	16.5	
	17	15.6	
	17	14.0	
	13	12.9	
	14	12.1	
	13	11.3	0.729
**#5 (back)**	13	13.4	
	13	15.5	
	14	7.3	
	14	6.9	
	15	12.5	
	15	16.7	
	16	16.0	
	16	15.5	
	16	15.0	
	17	14.3	
	18	14.1	
	18	13.5	0.311
**#2 (thigh)**	16	14.2	
	16	20.0	
	16	16.6	
	17	15.3	
	17	14.8	
	18	16.0	
	18	15.6	-0.313
**#6 (thigh)**	16	17.3	
	17	17.1	
	17	18.4	
	18	18.3	0.584
**#7 (thigh)**	11	8.6	
	12	10.9	
	13	10.7	
	14	13.3	
	14	14.3	
	15	15.6	
	16	18.8	
	17	20.9	
	17	19.0	0.978
**#8 (thigh)**	12	11.8	
	13	21.3	
	15	13.8	
	16	12.6	
	16	10.5	
	17	13.5	
	17	13.6	
	17	13.6	-0.345
**#9 (thigh)**	17	10.4	
	18	10.5	
	18	14.9	
	18	46	0.816

Table S9. Correlations between the Borg scale and the blood lactate for each subject.

Subjects	Borg scale	Lactate / mM	Pearson Coefficient
**#1 (back)**	15	2.6	
	16	3.3	
	16	4.2	
	16	5.0	
	14	3.4	
	12	2.0	
	12	1.6	
	12	1.3	0.881
**#3 (back)**	10	2.0	
	12	1.8	
	13	1.8	
	15	1.8	
	16	2.4	
	16	1.8	
	17	2.4	
	17	2.2	
	17	3.0	0.579
**#4 (back)**	15	3.6	
	16	4.2	
	17	3.7	
	17	4.1	
	17	4.7	
	17	5.0	
	13	3.1	
	14	2.2	
	13	1.9	0.853
**#5 (back)**	13	1.0	
	13	0.8	
	14	0.9	
	14	1.2	
	15	1.8	
	15	2.1	
	16	1.9	
	16	2.0	
	16	2.1	
	17	2.2	
	18	3.4	
	18	3.6	0.942
**#2 (thigh)**	16	3.2	
	16	4.0	
	16	4.7	
	17	5.6	
	17	5.3	
	18	5.6	
	18	6.7	0.864
**#6 (thigh)**	16	4.3	
	17	4.9	
	17	6.8	
	18	6.6	0.765
**#7 (thigh)**	11	1.7	
	12	1.6	
	13	1.7	
	14	2.5	
	14	2.3	
	15	2.6	
	16	3.2	
	17	3.9	
	17	4.2	0.954
**#8 (thigh)**	12	1.8	
	13	1.8	
	15	1.8	
	16	2.4	
	16	1.8	
	17	2.4	
	17	2.2	
	17	3.0	0.692
**#9 (thigh)**	17	2.1	
	18	3.1	
	18	3.0	
	18	3.4	0.961

3. Figures

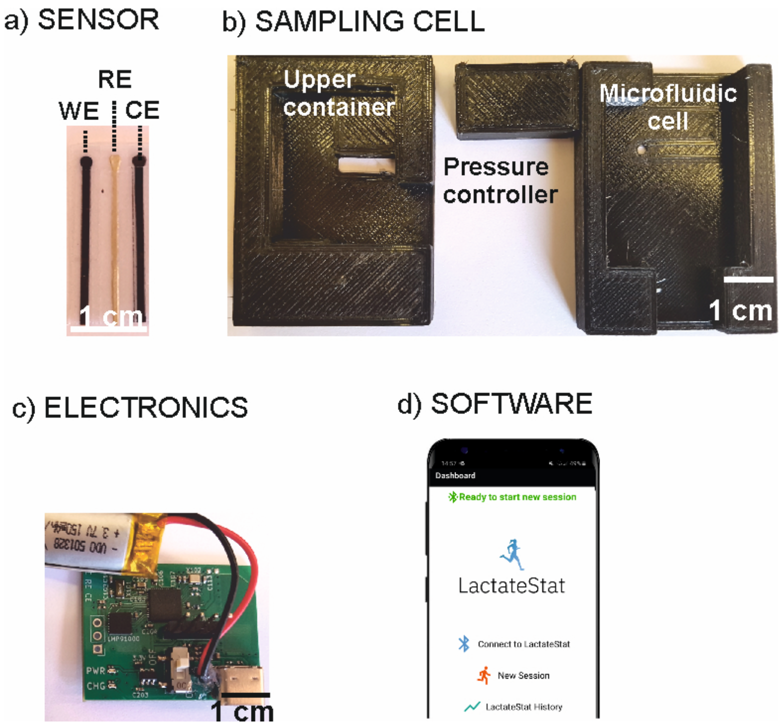

Figure S1. Pictures of (a) lactate sensor (consists of the WE, RE, and CE), (b) sampling cell, (c) electronics, and (d) software.

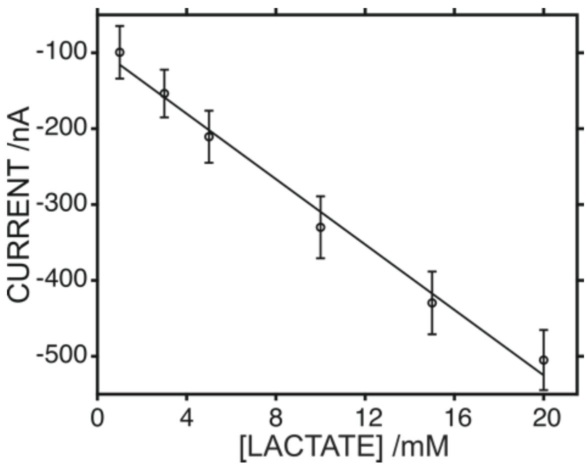

Figure S2. Average calibration curve observed in the reproducibility study (three twin sensors).

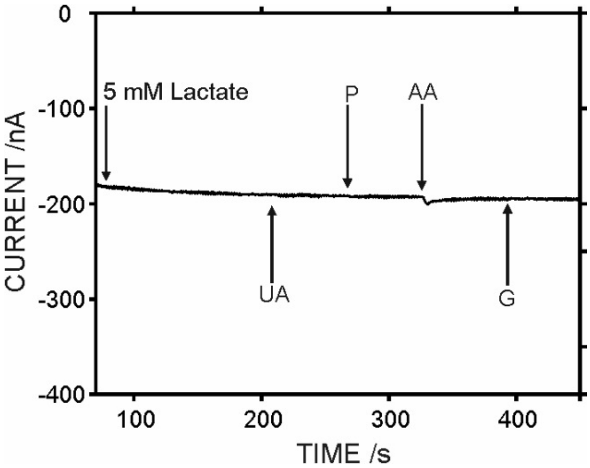

Figure S3. Interferences' study. The order of injections in artificial sweat background was: 5 mM lactate, 100 μM uric acid (UA), 100 μM pyruvate (P), 100 μM ascorbic acid (AA) and 250 μM glucose (G).

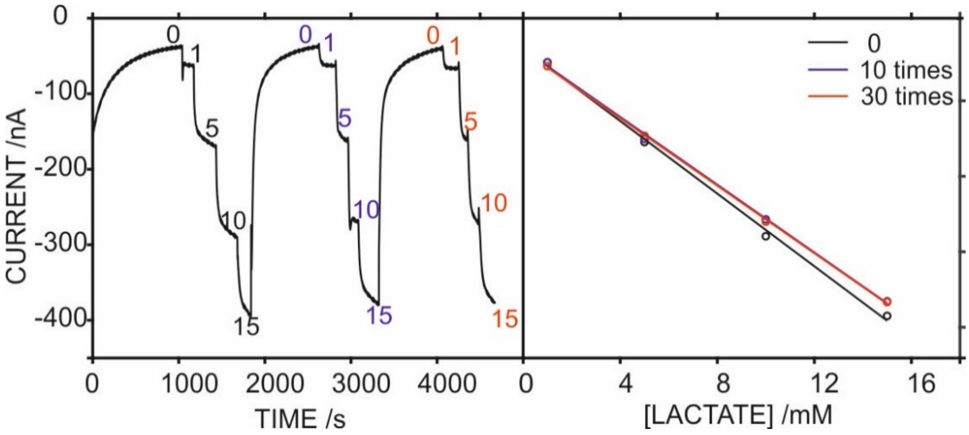

Figure S4. Resiliency test. Calibration graphs after the application of an increasing number of torsion strains. *Left:* Dynamic plots at different concentrations of lactate (the blue and red color of numbers represent the injection after 10 and 30 times, respectively). *Right:* Corresponding calibration curve.

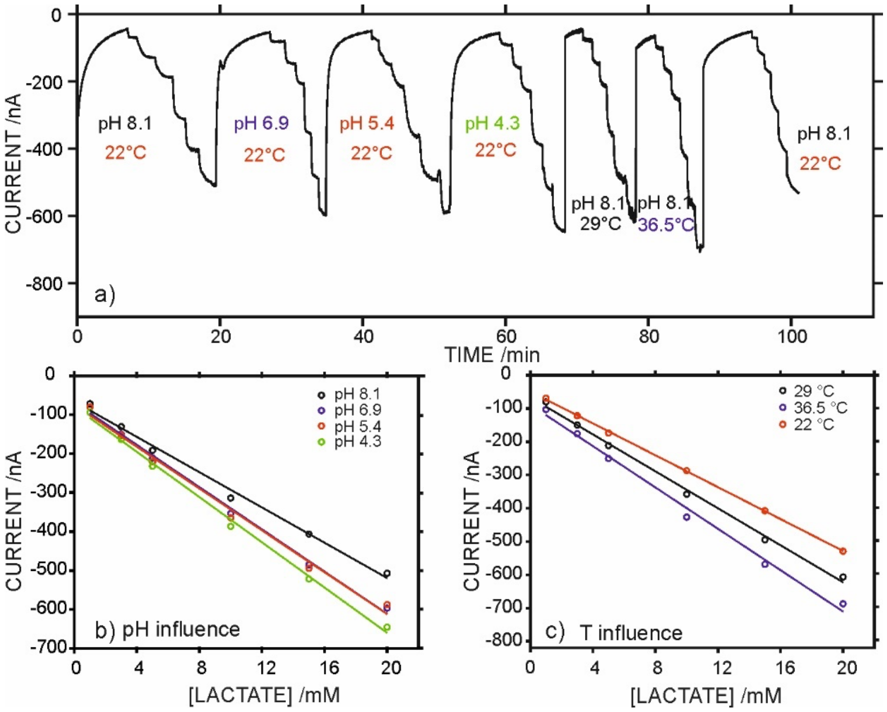

Figure S5. (a) Dynamic calibrations observed at varying pH and T conditions. (b,c) Corresponding calibration graphs.

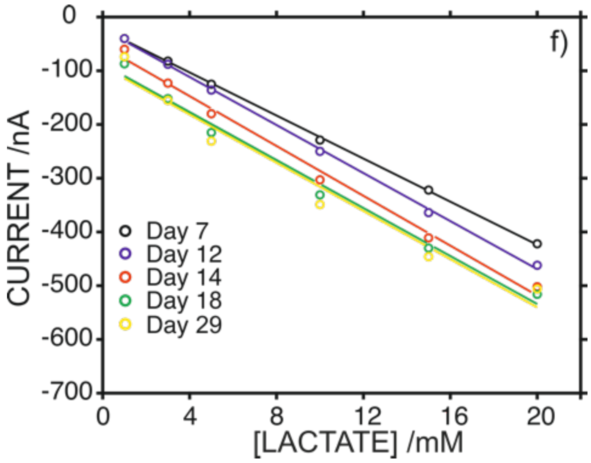

Figure S6. Lifetime investigation. Calibration graphs observed in different days from the preparation of the lactate biosensors.

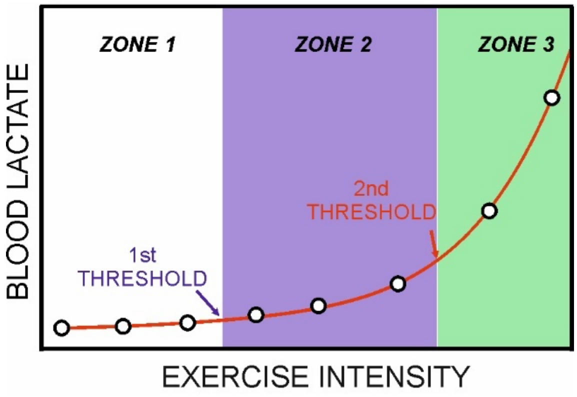

Figure S7. Schematics of the differentiation of zones 1, 2 and 3 in the training depending on the blood lactate thresholds. As a general trend, the first (aerobic) and second (anaerobic) lactate thresholds depend on low, moderate, and high exercise intensity.

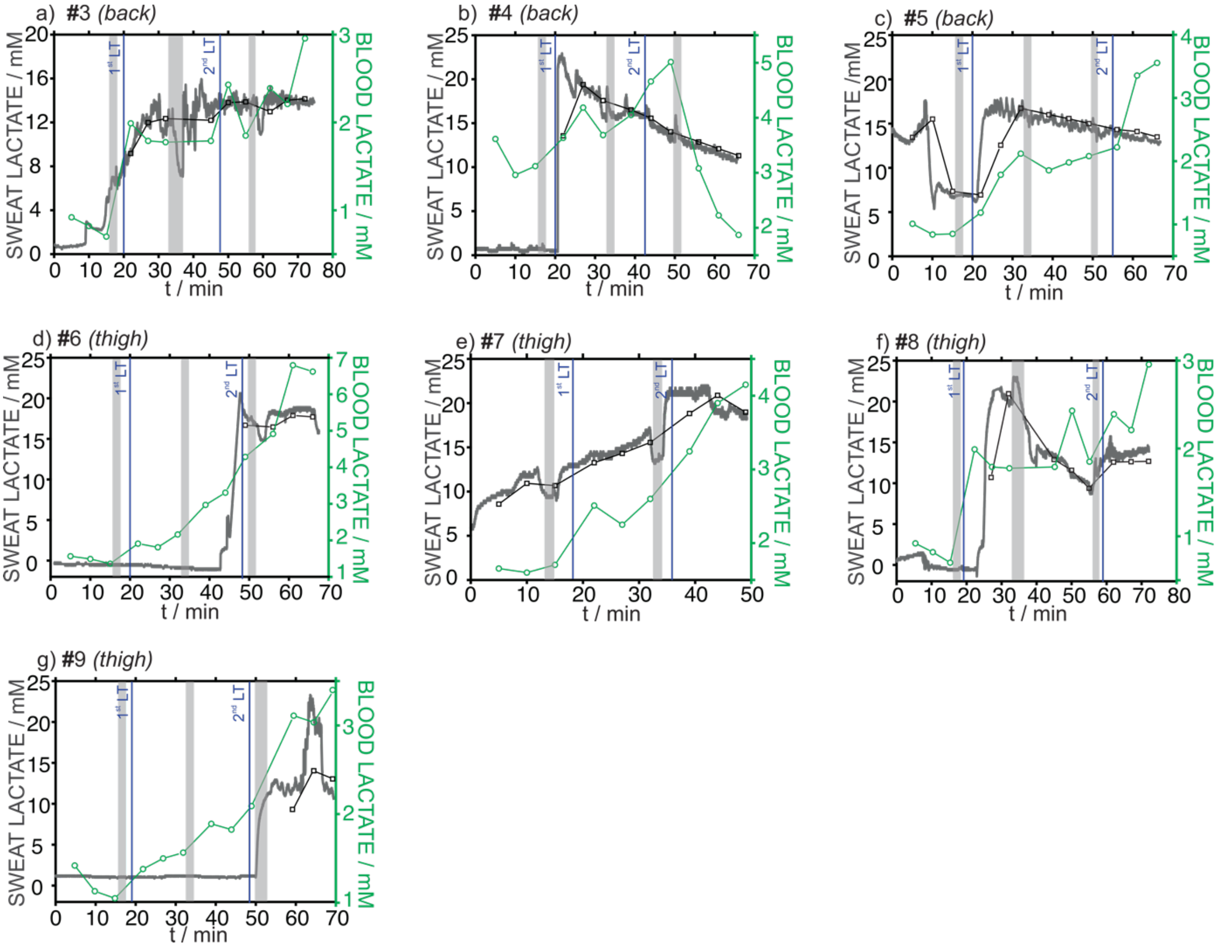

Figure S8. Real-time sweat lactate profiles measured during on-body tests in Dalarna with the wearable device attached to the back or thigh. Sweat and blood lactate were additionally analyzed in samples. Gray squares indicate the resting periods. Blue lines indicate the 1st and 2nd LT according to the blood lactate levels.

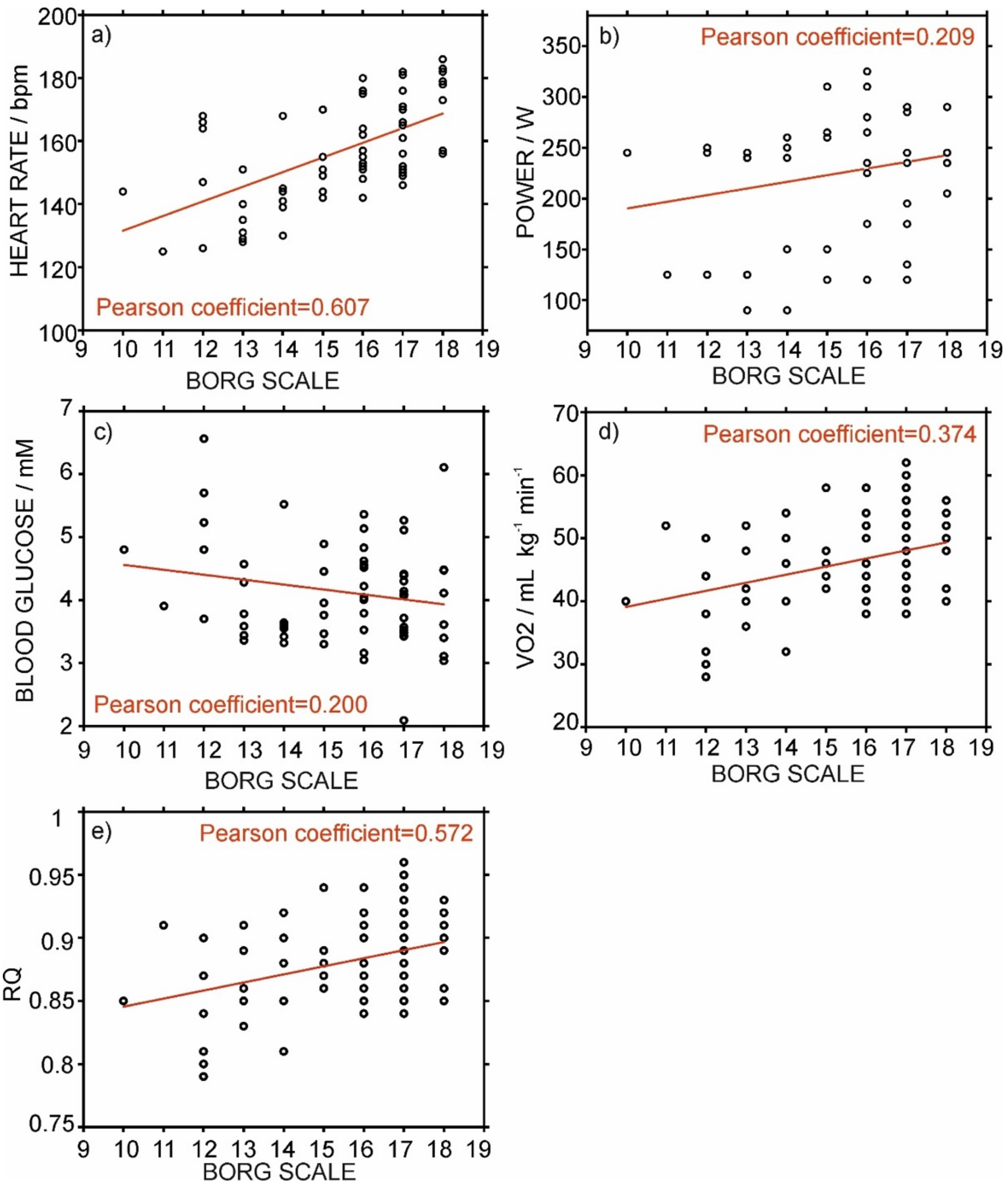

Figure S9. Correlations for the (a) heart rate, (b) power, (c) blood glucose, (d) VO_2, and (e) respiratory quotient with Borg scale. 70 samples obtained in Dalarna.

4. Outcomes and interpretations of the on-body tests regarding a possible relationship between sweat and blood lactate

Subject #3 (Figure S8a). From 25 to 45 min, the concentration of sweat lactate remained relatively constant at an average value of 12.2 ± 0.2 mM. A sudden decrease in sweat lactate concentration was observed around 35 min, which may be attributed to the resting period. From 50 min until the end of the training, a slightly higher lactate concentration was observed, with an average value of 13.8 ± 0.4 mM, which may be attributed to the slightly higher intensity of training during this period. The blood lactate concentration remained rather constant for the first 15 min, increased from the 20 min and it kept constant ay an average value of 1.8 ± 0.09 mM until 45 min (corresponding to the second zone). In the third zone (50 – 75 min), an increase in the blood lactate concentration up to 3.0 mM was observed. The ratio between sweat and blood lactate in the second zone was 6.7 – 7.0 times, and a mean value of 5.9 times in the third zone.

Subject #4 (Figure S8b). The sweat lactate concentration decreased between 20 and 30 minutes and stayed constant (15.9 ± 1.3 mM) between 35 and 50 min, corresponding to the second and third zones, respectively. The subsequent drop in sweat lactate concentration down to a value of 11.3 mM was attributable to the decreasing of training intensity, which also led to a decrease in blood lactate concentration. Blood lactate concentration started at 3.6 mM and gradually increases up to 5.0 mM (at 50 min). Then, the blood lactate drops down to 1.8 mM due to the lowering intensity. The ratio between sweat and blood lactate in the second and third zones were 2.8 – 4.0 and 3.7 – 4.8 times, respectively. After that, the ratio was 4.2 – 6.1 times while blood lactate back to 1.8 mM.

Subject #5 (Figure S8c). The dynamic profile for sweat lactate was slightly different from the other three subjects. The sweat lactate decreased from ca. 14 mM to 7.1 mM before reaching the first lactate threshold, maintaining a constant value of 7.1 ± 0.2 mM while blood lactate showed around 1.0 mM. Then, sweat lactate increases up to 16.5 mM at 21 min and likely maintained a constant value of 15.0 ± 1.0 mM in the second and third zones. Apart from the dilution period (sweat and blood ratio is 5.8 – 8.6 times, 10 – 21 min), the ratio of sweat and blood lactate showed relatively high values in the range of 13.3 – 18.4 in the first zone. The sweat lactate in the second and third zones were 7.0 – 8.7 and 3.7 – 4.2 times higher than blood lactate, respectively.

Subject #6 (Figure S8d). During the first 45 min of the test, the sweat lactate concentration was not measured due to the low perspiration rate, which commonly happened in the on-body test with the thigh part. From the point where the second lactate threshold appears (48 min), a consistent sweat lactate value of 17.8 ± 0.6 mM was noted, representing a concentration 2.7 – 4.2 times higher than the corresponding level of lactate in the blood.

Subject #7 (Figure S8e). A notable increase in sweat lactate from 5.3 mM to approximately 21 mM was observed, with a relatively constant level of 19.5 ± 0.9 mM evident during the training period of 35 – 50 min. A slight decrease in sweat lactate between each training round was noted, likely due to the resting period when the participant was not actively exercising. Blood lactate remained constant during 5 – 15 min and subsequently rose to 2.5 mM, which represented the second zone (20 – 25 mi). After that, blood lactate exhibited a continuous increase from 35 min onwards, corresponding to the second lactate threshold, and ultimately

peaked at 4.6 mM at the end of the test. Of note, sweat lactate shows a 5.2 – 6.8, 5.2 – 6.4 times, 4.6 – 5.8 times higher than blood lactate in the first, second, and third zone, respectively.

Subject #8 (Figure S8f). From 22 min after initiating test, the lactate concentration of sweat was observed at the highest value of 22.5 mM. Then, the lactate decreased down to 10.5 mM between 34 – 56 min. From third zone, the lactate concentration increased to 13.5 mM and maintained a constant afterwards (13.5 ± 0.04 mM) until end of test. The sweat lactate was 5.5 – 12.0 and 4.5 – 5.7 times higher than blood lactate in the second and third zones, respectively.

Subject #9 (Figure S8g). An extended interval was observed for the first detection of sweat lactate, approximately at 50 min, due to a low perspiration rate on the thigh. Upon the manifestation of the second lactate threshold, the concentration of sweat lactate started at 10.4 mM and persisted at this level with minor fluctuations for a brief duration before experiencing a rapid increase to 22.5 mM, probably due to the high concentration of blood lactate. In the third zone, the concentration of sweat lactate was found to be 3.4-4.9 times higher than that of the corresponding blood lactate.

5. References

(1) Xuan, X.; Pérez-Ràfols, C.; Chen, C.; Cuartero, M.; Crespo, G. A. Lactate biosensing for reliable on-body sweat analysis. *ACS sensors* **2021**, *6* (7), 2763-2771.
(2) Jia, W.; Bandodkar, A. J.; Valdés-Ramírez, G.; Windmiller, J. R.; Yang, Z.; Ramírez, J.; Chan, G.; Wang, J. Electrochemical tattoo biosensors for real-time noninvasive lactate monitoring in human perspiration. *Analytical chemistry* **2013**, *85* (14), 6553-6560.
(3) Yokus, M. A.; Saha, T.; Fang, J.; Dickey, M. D.; Velev, O. D.; Daniele, M. A. Towards Wearable Electrochemical Lactate Sensing using Osmotic-Capillary Microfluidic Pumping. In *2019 IEEE SENSORS*, 2019; IEEE: pp 1-4.
(4) Gao, W.; Emaminejad, S.; Nyein, H. Y. Y.; Challa, S.; Chen, K.; Peck, A.; Fahad, H. M.; Ota, H.; Shiraki, H.; Kiriya, D. Fully integrated wearable sensor arrays for multiplexed in situ perspiration analysis. *Nature* **2016**, *529* (7587), 509-514.
(5) Saha, T.; Songkakul, T.; Knisely, C. T.; Yokus, M. A.; Daniele, M. A.; Dickey, M. D.; Bozkurt, A.; Velev, O. D. Wireless wearable electrochemical sensing platform with zero-power osmotic sweat extraction for continuous lactate monitoring. *ACS sensors* **2022**, *7* (7), 2037-2048.
(6) Zhang, Q.; Jiang, D.; Xu, C.; Ge, Y.; Liu, X.; Wei, Q.; Huang, L.; Ren, X.; Wang, C.; Wang, Y. Wearable electrochemical biosensor based on molecularly imprinted Ag nanowires for noninvasive monitoring lactate in human sweat. *Sensors and Actuators B: Chemical* **2020**, *320*, 128325.
(7) Li, M.; Wang, L.; Liu, R.; Li, J.; Zhang, Q.; Shi, G.; Li, Y.; Hou, C.; Wang, H. A highly integrated sensing paper for wearable electrochemical sweat analysis. *Biosensors and Bioelectronics* **2021**, *174*, 112828.
(8) Jiang, D.; Xu, C.; Zhang, Q.; Ye, Y.; Cai, Y.; Li, K.; Li, Y.; Huang, X.; Wang, Y. In-situ preparation of lactate-sensing membrane for the noninvasive and wearable analysis of sweat. *Biosensors and Bioelectronics* **2022**, *210*, 114303.
(9) Komkova, M. A.; Eliseev, A. A.; Poyarkov, A. A.; Daboss, E. V.; Evdokimov, P. V.; Eliseev, A. A.; Karyakin, A. A. Simultaneous monitoring of sweat lactate content and sweat secretion rate by wearable remote biosensors. *Biosensors and Bioelectronics* **2022**, *202*, 113970.
